# The N6-Methyladenosine Modification and Its Role in mRNA Metabolism and Gastrointestinal Tract Disease

**DOI:** 10.3389/fsurg.2022.819335

**Published:** 2022-01-28

**Authors:** Teng Cai, Lawrence Lawer Atteh, Xianzhuo Zhang, Chongfei Huang, Mingzhen Bai, Haidong Ma, Chao Zhang, Wenkang Fu, Long Gao, Yanyan Lin, Wenbo Meng

**Affiliations:** ^1^The First Clinical Medical College, Lanzhou University, Lanzhou, China; ^2^Gansu Province Key Laboratory Biotherapy and Regenerative Medicine, Lanzhou, China; ^3^The Department of General Surgery, The First Hospital of Lanzhou University, Lanzhou, China; ^4^Gansu Province Institute of Hepatopancreatobiliary Surgery, Lanzhou, China

**Keywords:** N6-methyladenosine, mRNA metabolism, gastrointestinal tract, tumor, non-tumor diseases

## Abstract

The N6-methyladenosine (m6A) modification is the most abundant internal modification of messenger RNA (mRNA) in higher eukaryotes. Under the actions of methyltransferase, demethylase and methyl-binding protein, m6A resulting from RNA methylation becomes dynamic and reversible, similar to that from DNA methylation, and this effect allows the generated mRNA to participate in metabolism processes, such as splicing, transport, translation, and degradation. The most common tumors are those found in the gastrointestinal tract, and research on these tumors has flourished since the discovery of m6A. Overall, further analysis of the mechanism of m6A and its role in tumors may contribute to new ideas for the treatment of tumors. m6A also plays an important role in non-tumor diseases of the gastrointestinal tract. This manuscript reviews the current knowledge of m6A-related proteins, mRNA metabolism and their application in gastrointestinal tract disease.

## Introduction

Epigenetics refers to heritable changes in gene expression or cell phenotypes that usually occur without alteration of the DNA sequence but rather result in slight or substantial modifications to DNA or RNA. To date, more than 100 types of RNA modifications, including mRNA, tRNA, rRNA, and lncRNA modifications, have been discovered (1). The most common modification of RNA is methylation, and the N6-methyladenosine (m6A) modification is the most common modification of mammalian mRNA (2).

m6A is a posttranscriptional modification through which a methyltransferase methylates the 6th nitrogen atom of adenine (A). m6A was first detected on mRNA in hepatocellular carcinoma cells (3) and later on non-coding RNAs such as miRNA (4), circRNA (5), lncRNA (6), and snRNA (7). Similar to the results observed with DNA methylation, the m6A modification of mRNA is a reversible and dynamic modification process catalyzed by the actions of methylases and demethylases. The rapid development of immune coprecipitation and RNA sequencing technology in recent years has led to great progress in research on the m6A modification of mRNA, which have led to a deeper understanding of the mechanism of m6A and its role, particularly in tumors. In this paper, the relationship between m6A-related proteins, mRNA metabolism and digestive system tumors is described in detail.

## Discovery and Distribution of m6A

The m6A modification of mRNA is a posttranscriptional modification in which the 6th nitrogen atom of adenine is methylated by a methyltransferase (8). This modification can affect the stability, splicing, transport, nucleation and translation of mRNA (9–12). m6A was first discovered in bacteria by Dunn and Smith (13). In 1974, Desrosiers et al. (3) observed the methylation of mRNA in liver cancer cells and found that m6A accounted for ~80% of modifications and could be regarded as the most important mode of methylation. In general, m6A can be found on many eukaryotic (14–18) and viral (19–21) mRNAs. Although m6A was discovered early and is widely distributed, the related research progress has been slow due to technical limitations. However, the presence of m6A has gained recognition, and two studies in 2012 (8, 9) detected more than 12,000 m6A sites in human and mouse cells, including more than 7,000 types of mRNAs and more than 300 types of lncRNAs. m6A mainly clusters around stop codons, 3′ untranslated regions (3′ UTRs) and internal long exons of mRNA, and the sites are highly conserved. In other words, m6A modification mainly occurs in the highly conserved sequence RRACH (R = G or A; H = A, C, or U). This modification also occurs on other RNAs, such as tRNA and rRNA, but the related conserved sequence is different from that found for mRNA (9). In addition to the type of RNA, the distribution of m6A is specific in human tissues. For example, the content of m6A in the liver, kidney and brain is significantly higher than that in other tissues (8), which indicates that m6A may also play a vital role in the differentiation and development of tissues and organs. Furthermore, m6A expression varies among cancer cell lines and is closely related to the self-renewal of tumor stem cells in tumorigenesis (22).

## m6A-Related Enzymes

**Figure 1:**
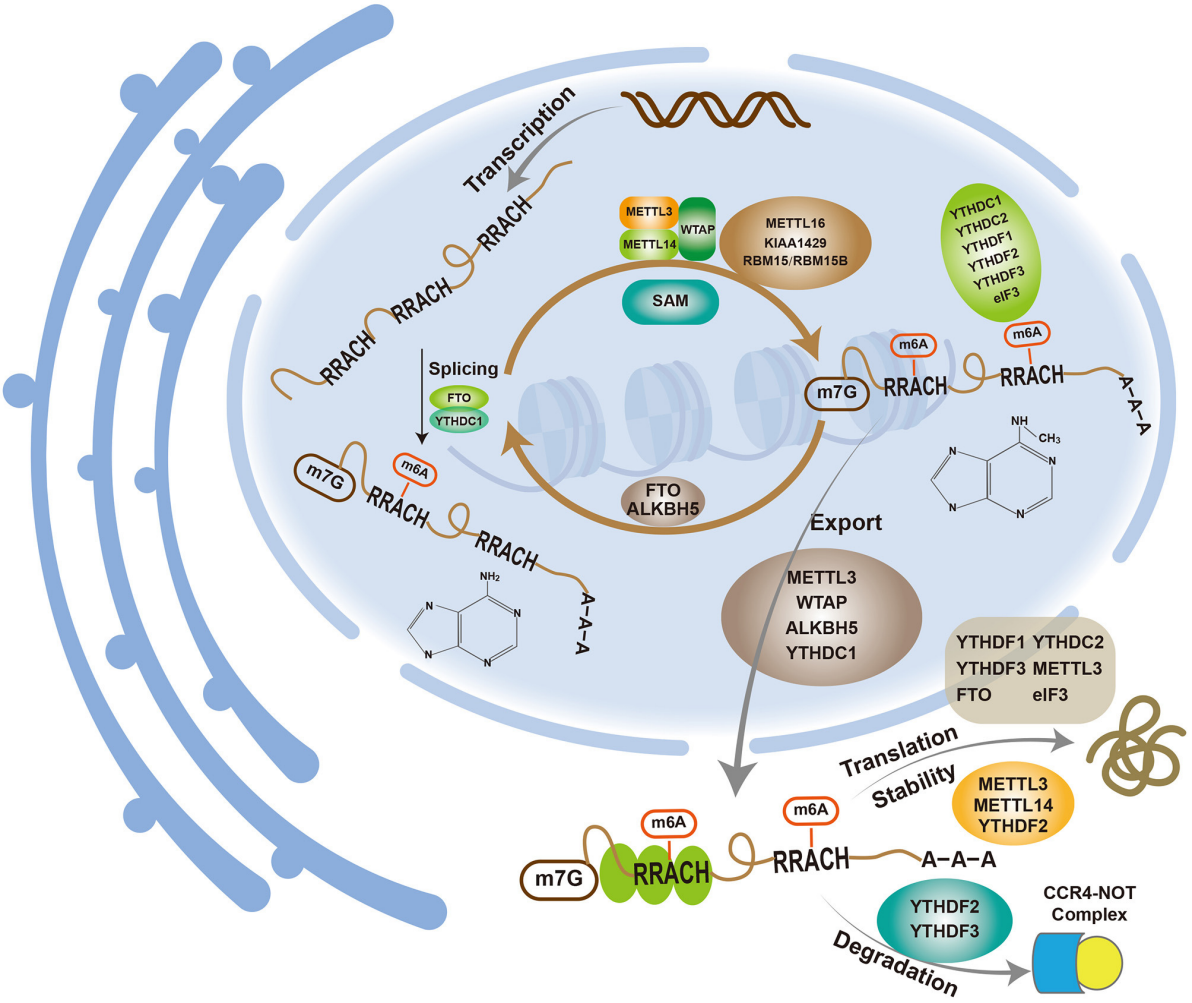
m6A participates in various metabolic processes related to mRNAs.

The discovery of fat mass and obesity-associated protein (FTO), a type of m6A demethylase (23), revealed that the m6A modification of mRNA is a dynamic and reversible process influenced by three enzymes: methyltransferases (“writers”), demethylases (“erasers”) and methyl binding proteins (“readers”) (Figure 1).

### m6A Writers

The m6A methyltransferase complex comprises methyltransferase-like 3 (METTL3), methyltransferase-like 14 (METTL14) and Wilms tumor 1-associated protein (WTAP). The complex utilizes S-adenosylmethionine (SAM) as the methyl group donor to methylate the 6th nitrogen atom of adenine to form m6A. METTL3, which is also called MT-A70, was the earliest discovered m6A methyltransferase and was originally isolated and purified from HeLa cells (24). METTL3 has SAM-binding activity and is highly conserved (25, 26). Because almost all m6A methylation modifications are lost after METTL3 knockdown (27), METTL3 is a key component of the m6A methyltransferase complex and the only methyltransferase that is currently known to bind to SAM. METTL14, another member of the methyltransferase complex, showed high (43%) homology to METTL3 (28). As observed in previous studies, the knockdown of METTL14 in HeLa cells decreases the m6A methylation level; thus, METTL14 is another important component of the m6A methyltransferase complex (28). Although METTL14 exhibits high homology with METTL3, it lacks a SAM-binding region and thus cannot bind to SAM. METTL14 is mainly responsible for the identification and localization of subunits, whereas METTL3 has catalytic activity (28). METTL3 and METTL14 interact at a 1:1 ratio to form a stable methyltransferase complex and catalyze the m6A modification of mRNA *in vivo* (29). Moreover, *in vitro* experiments have shown that METTL14 exhibits higher enzyme activity than METTL3, and their heterodimer exhibits markedly enhanced enzyme activity than either enzyme alone, which indicates that the two enzymes play a synergistic role in the methylation process (28, 30–32). WTAP is the third most important component of the methyltransferase complex. Similar to METTL14, WTAP lacks a SAM-binding region and has no catalytic activity. However, the knockdown of WTAP results in the absence of METTL3 and METTL14 at nuclear speckles and decreases the m6A level. Therefore, WTAP may colocalize at nuclear speckles by recruiting the METTL3-METTL14 heterodimer to promote the m6A modification of mRNA (33). In recent years, many new methyltransferase components, such as KIAA1429, RBM15/RBM15B, and METTL16, have been identified. KIAA1429 is mainly involved in mRNA 3′UTR and stop codon methylation, and its silencing reduces the m6A levels (34). Another study showed that interfering with the expression of KIAA1429 in lung cancer cells decreases the level of m6A, and the effect was more obvious than that observed with METTL3 or METTL14. Nevertheless, the role of KIAA1429 in the methyltransferase complex remains unclear (35). RBM15/RBM15B can interact with METTL3 and bind to the U-rich region near the m6A modification site to recruit methyltransferases to a methylation site, which requires the participation of WTAP (36, 37). METTL16, a newly discovered methyltransferase, correlates positively with m6A expression and is mainly involved in the methylation modification of U6 nucleolar RNA (U6 snRNA) and methionine adenosine transferase 2A (MAT2A) mRNA (7, 38).

### m6A Erasers

The main m6A demethylases are FTO and AlkB homolog 5 (ALKBH5). FTO is a member of the Fe(II)- and α-ketoglutarate-dependent dioxygenase ALKB protein family, which is widely found in human tissues and primarily involved in the regulation of fat and energy metabolism (39). FTO also plays an important role in diabetes, cardiovascular diseases and tumors (40, 41). In 2011, FTO was proven to be a demethylase involved in the demethylation of m6A (23). Indeed, the knockdown of FTO increases the m6A level but does not affect the expression of METTL3, which indicates an independent modification of m6A by FTO. Overall, the m6A modification of mRNA is a reversible and dynamic process, and a new era of m6A research has begun. ALKBH5, the second discovered demethylase, also belongs to the ALKB family, and its expression is negatively correlated with the m6A modification of mRNA (42). Unlike other members of the family, ALKBH5 only demethylates m6A on single-stranded RNA/DNA (43). Although ALKBH5 and FTO are homologous, they act independently, do not interfere with each other and exhibit some differences. ALKBH5 is mainly localized to the nucleus and can directly alter the m6A levels via demethylation. Most FTO is located in nuclear speckles, and hm6A and fm6A, which are known as intermediates, is needed for m6A demethylation by FTO (44). ALKBH5 is mainly expressed in the testis and is involved in sperm formation (42), whereas FTO exists in a wide range of human tissues but is mainly expressed in the brain. In addition to its effect on m6A, FTO demethylates m6Am, and its activity on m6Am is 100 times higher than that on m6A. The real substrate of FTO may be m6Am rather than m6A (45).

### m6A Readers

In addition to methyltransferase and demethylase, the m6A modification of mRNA requires the involvement of methyl-binding proteins that recognize m6A sites. The readers identified to date mainly include YTH domain proteins, eukaryotic translation initiation factor 3 (eIF3) and human heterogeneous nuclear ribonucleoprotein A2B1 (HNRNPA2B1). The YTH domain proteins are further divided into the DC (YTHDC1 and YTHDC2) and DF (YTHDF1, YTHDF2, and YTHDF3) families. The three YTHDF proteins exhibit a similar structure, are mainly distributed in the cytoplasm, and can bind to all m6A sites on mRNA (9, 11). Among these proteins, YTHDF2 was the first reader found to degrade m6A RNA through the CCR4-NOT complex (46). YTHDF1 is involved in protein translation, but the process requires eIF3 and other factors (12). Although the regulatory function of YTHDF3 remains unclear, it has been reported to act in synergy with YTHDF1 to promote the translation of methylated RNA and synergizes with YTHDF2 to accelerate mRNA decay (47, 48). YTHDC is mainly distributed in the nucleus. YTHDC1 is involved in the modification of immature mRNA, and some nuclear non-coding RNAs regulate mRNA splicing and mediate the transfer of m6A mRNA from the nucleus to the cytoplasm (49, 50). YTHDC2 can improve the target translation efficiency and reduce the mRNA abundance (51). Studies have also shown that YTHDC2 can promote colon cancer metastasis through HIF-1 and is a potential diagnostic and therapeutic target of this tumor (52). eIF3 mediates translation initiation by binding to the 5′UTR of m6A mRNA in two ways: direct binding and indirect binding through YTHDF (12, 53). HNRNPA2B1, which is mainly located in the nucleus, recognizes m6A sites on precursor miRNAs through interaction with DGCR8, participates in the splicing and processing of precursor miRNAs and thus regulates the generation of mature miRNAs (54).

## m6A and mRNA

m6A is involved in mRNA metabolism (Figure 1).

### m6A Influences mRNA Maturation

mRNA maturation includes 5′-capping, 3′-tailing and intron splicing. More m6A sites are found on premRNA than on mature mRNA, which indicates that introns also contain m6A sites. The m6A modification mainly occurs in the nucleus with the splicing of introns, which leads to a reduction in the number of m6A sites on mature mRNA (55). Many m6A modification-related proteins, writers (such as METTL3, METTL14, and WTAP), erasers (such as FTO and ALKBH5) and readers (YTHDC1), are primarily found in nuclear speckles (23, 33, 42, 49, 56). As mentioned above, the m6A modification may occur mainly in the nucleus, where m6A plays a role in mRNA splicing. The knockdown of METTL3 in mouse embryonic stem cells results in exon hopping and intron retention splicing abnormalities (27). Serine/arginine-rich splicing factor 2 (SRSF2) is an important splicing factor. Zhao et al. found that the knockdown of FTO in 3T3-L1 preadipocytes increases the m6A level in premRNA, which further promotes the binding ability to SRSF2 and results in an increase in target exons (57). FTO regulates differentiation by regulating the m6A levels around splice sites to control exon splicing of the adipogenic regulator RUNX1T1. YTHDC1 can block the binding of SRSF10 (SRp38) to mRNA by recruiting SRSF3 (SRp20), promoting an increase in the exons of targeted mRNA and thus aiding the selective splicing of mRNA (49). Dominissini et al. (9) found that the knockout of METTL3 reduces the level of m6A on mRNA and also decreases the level of gene expression through effects on the p53 signaling pathway and apoptosis. Thus, m6A plays an important role in mRNA splicing and promotes mRNA maturation through splicing.

### m6A Affects mRNA Export

Gene expression involves transcription, i.e., mRNA synthesis, and translation, i.e., protein synthesis, which utilize DNA and mRNA as templates, respectively. The connection of the two processes requires the transfer of mRNA from the nucleus to the cytoplasm, which is a process termed export. Changes in export, including enhancement and suppression, alters gene expression. m6A can affect the export of mRNA. Fustin et al. found that silencing the m6A methylase METTL3 inhibits m6A methylation, which suppresses the export of mRNA and delays its processing (58). ALKBH5 also affects the export of mRNA, and its knockdown enhances the process (42).

### m6A Affects mRNA Translation

There is no unified conclusion regarding the effect of m6A on mRNA translation: m6A can promote or inhibit translation or may have no effect. Earlier studies have suggested that the translational effect of m6A-containing mRNA is 1.5 times greater than that of m6A-free mRNA (59). METTL3 can improve the translation of a target mRNA by recruiting eIF3 to the translation initiation complex, and this process is independent of its methyltransferase activity (60). Additionally, YTHDF1 promotes mRNA translation with eIF3 participation (12), although m6A also reportedly decreases mRNA translation (61). Hess et al. found that the knockdown of FTO in mice lead to an increase in m6A and a significant increase in mRNA, and the corresponding protein levels increase, decrease or show no significant change (62). The reasons for these observations remain unclear but may be related to tissue specificity or m6A sites, and further study is needed.

### m6A Regulates mRNA Stability

m6A can affect not only the splicing, translation and export of mRNA but also its stability. Dominissini et al. found that the knockdown of METTL3 decreased the m6A mRNA and gene expression levels, which indicates that m6A promotes the stability of mRNA (9). However, Wang et al. studied mouse embryonic stem cells and found that m6A is negatively correlated with mRNA stability (32). The knockdown of METTL3 and METTL14 decreases the level of mRNA m6A, which promotes the binding of human antigen R (HuR) to mRNA and thus increases its stability. Xie et al. (63) found that METTL3 could induce the downregulation of BATF2 expression in gastric cancer (GC) because METTL3 catalyzes the m6A modification of BATF2 mRNA, which reduces mRNA stability. Yan et al. (64) also reported that METTL3 can reduce the stability of PTEN mRNA and thus promotes the proliferation, migration and invasion of GC cells. Interestingly, METTL3 reportedly increases mRNA stability in GC. Wang et al. (65) found that H3K27 could acetylate METTL3 to increase its expression in GC, and this increased METTL3 level could induce the m6A modification of HDGF mRNA to increase its mRNA stability. HDGF promotes tumor growth and liver metastasis by promoting tumor angiogenesis. In addition, METTL3 can increase its stability through the m6A modification of ARHGAP5 mRNA in GC cells, which results in drug resistance (66). In colorectal cancer (CRC), Sec62 binds to β-catenin to inhibit its degradation and enhance WNT signaling, which leads to increased stemness and chemoresistance in CRC cells. The increase in Sec62 is caused by METTL3 increasing the stability of its mRNA (67). YTHDF2 also affects mRNA stability. Wang et al. reported that the C-terminal domain of YTHDF2 selectively binds to mRNA-containing m6A but that the N-terminal domain is responsible for binding to the YTHDF2-mRNA complex and directing it to the cellular RNA decay site for mRNA degradation (11). The knockdown of YTHDF2 increases the mRNA stability and prolongs the lifespan. Du et al. also confirmed that YTHDF2 can reduce the stability of m6A mRNA through the CCR4-NOT complex, which leads to its degradation (46). In CRC, YTHDF2 can increase the m6A modification of GSK3β mRNA, reduce the stability of GSK3β mRNA, and promote its degradation, which induces CRC cell proliferation and tumor progression (68). In addition, insulin-like growth factor 2 mRNA-binding proteins (IGF2BPs) dynamically promote RNA stability and/or increase mRNA storage under different physiological conditions (69). The above-mentioned studies indicate that the regulation of mRNA stability by m6A is not merely related to the cell type; that is, the same m6A-related protein may play opposite roles in different cells, and these different roles may also be related to external factors.

## m6A and Gastrointestinal Tract Tumors

### Hepatocellular Carcinoma and Cholangiocarcinoma

Digestive system tumors are the most common types of tumors, and many m6A-related proteins play important roles in the development and onset of these tumors. As the core component of the m6A methyltransferase complex, METTL3 is significantly increased in HCC tissues and associated with the clinical aspects of tumors (70). METTL3 inhibits the expression of SOCS2 through an m6A-YTHDF2-dependent mechanism and thus plays a role in HCC.

The knockdown of METTL3 inhibits HCC growth and metastasis, whereas its overexpression has the opposite effect. Interestingly, another study found that the m6A levels in normal liver tissues, adjacent normal liver tissues, and HCC tissues are successively reduced, which indicates that the m6A levels decrease from normal tissues to HCC tissues and that a low level of m6A is mainly associated with the downregulation of METTL14 (71). In addition to the occurrence of HCC, METTL14 downregulation is related to the downregulation of micRNA126 by interacting with DGCR8 to promote HCC metastasis. Cellular experiments have also demonstrated that METTL14 is negatively associated with HCC invasion and metastasis. This study also found that FTO is significantly downregulated in liver cancer tissues. A possible explanation is that METTL14 downregulation causes a decrease in the m6A levels and thereby leads to the downregulation of FTO to compensate for demethylation. Although m6A methylation appears to be a dynamic reversible process, there is no direct evidence. For the same tumor, two studies found that different m6A-related proteins play a major role, which may be due to sample error, and further investigation is thus needed. Another study found that YTHDF2 exhibits significantly higher expression in HCC tissues, whereas the change in YTHDF2 is negatively regulated by micRNA145 (72). In HepG2 cells, micRNA145 targets and binds to the 3′UTR of YTHDF2 mRNA, which causes a decrease in the YTHDF2 mRNA and protein levels and a decrease in the m6A levels. However, this study only found changes in the expression of the reading protein YTHDF2 in liver cancer and did not address the role of m6A in liver cancer. Moreover, only one liver cancer cell line, HepG2, was used, and no *in vivo* or *in vitro* experiments were performed. Regardless of these limitations, these two studies show that m6A-related proteins and miRNAs can interact and regulate each other and play a role in the occurrence and development of liver cancer. YTHDF1 has also been confirmed to be abnormally expressed in liver cancer, and its expression is upregulated in tumor tissues (73). Unfortunately, this study was based only on clinical data, and no *in vivo* and *in vitro* experiments were performed for verification; thus, the role of YTHDF1 in liver cancer needs to be further explored.

After primary liver cancer, CCA is the second most common malignant tumor of the hepatobiliary system, accounting for 10–15% of all hepatobiliary malignancies. Due to the absence of obvious symptoms at the early stage and the lack of specific diagnostic markers, most CCA cases are at an inoperable stage at the time of diagnosis (74). Although few studies have investigated m6A in CCA, a previous study showed that WTAP expression is increased in CCA tissues and that its overexpression or knockdown affects the metastatic ability of CCA. Nonetheless, this study failed to indicate whether the effect of WTAP on CCA is related to the m6A modification (75). The relationship between m6A and CCA is relatively clear with regard to FTO (76). FTO is significantly reduced in intrahepatic CCA and is associated with tumor differentiation and patient prognosis. The knockdown or overexpression of FTO decreases or increases the sensitivity of intrahepatic cholangiocarcinoma cells to cisplatin, respectively. FTO overexpression also inhibits tumor growth in mice. However, the study only examined intrahepatic CCA, excluded a large proportion of extrahepatic CCA cases, and did not elucidate the exact mechanism of action of FTO. Therefore, the role of m6A in cholangiocarcinoma remains unclear.

### GC

In recent years, a number of studies have shown that m6A, particularly METTL3, is closely related to the occurrence and development of GC. Wang et al. found that the expression of METTL3 is significantly increased in GC tissues and associated with poor prognosis (65). Through a process mediated by P300, H3K27 acetylates METTL3 to increase its expression, and increased METTL3 promotes the m6A methylation of the mRNA of the downstream protein HDGF to enhance its stability. HDGF promotes tumor growth and liver metastasis by promoting tumor angiogenesis on the one hand and activating the expression of GLUT4 and ENO2 on the other hand, which results in promotion of glycolysis in GC cells. This study also comprises the first investigation that combines m6A with glucose metabolism to study its role in GC. Su et al. found that most m6A methylation-related proteins (METTL3, METTL14, WTAP, KIAA1429, RBM15, ZC3H13, YTHDC1, YTHDC2, YTHDF1, YTHDF2, HNRNPC, and FTO) are more highly expressed in GC tissues than in normal tissues and that patients with poor prognosis exhibit higher FTO expression (77). Yang et al. observed higher levels of m6A in GC tissues than in adjacent tissues (78). Subsequently, these researchers detected methylases related to m6A and found that the mRNA levels of METTL3 and METTL14 are significantly increased but that WTAP, ALKBH5, and FTO did not exhibit significant changes. The role of METTL3 in the genesis and development of GC has been elucidated through its downstream targeting of the MYC pathway, and the results show that METTL3 acts as an oncogene in GC. However, both of these studies have shortcomings. The former study did not include any practical investigations, and all the results were based on database analysis and thus cannot be easily applied to clinical treatment. Although the latter study investigated the m6A modification of downstream targets, it did not specify which protein was regulated by m6A and did not evaluate the function of downstream targets. Liu et al. also confirmed that the m6A level is increased in GC tissues compared with adjacent tissues and found that METTL3 playa a major role among m6A-related proteins (79). METTL3 is elevated in tumor tissues and increases with progression of the tumor stage: a higher expression is associated with a worse prognosis. Cellular experiments have further demonstrated that METTL3 affects the proliferation and migration ability of GC cells by regulating the expression levels of GFI-1 and epithelial-mesenchymal transition (EMT). Xie et al. (63) found that BATF2 is a tumor suppressor in GC and exhibits significantly decreased mRNA and protein levels in GC tissues. However, the downregulation of BATF2 is due to the m6A modification of its mRNA by METTL3, which reduces its stability. This study combined the regulation of mRNA metabolism by m6A with its role in tumors and fundamentally illustrated the action of m6A in GC. In addition, Lin et al. (80) found that METTL3 can promote the proliferation, migration and invasion ability of GC cells by inhibiting apoptosis and activating the AKT pathway. Yue et al. (81) also reported that the expression of METTL3 is higher in GC tissues than in adjacent normal tissues and increases with progression of the tumor stage. Mechanistically, METTL3 affects the migration and invasion ability of GC cells by regulating the expression of zinc finger MYM-type containing 1 (ZMYM1) and promoting EMT. This process also involves the reader protein HuR. In addition to the role of m6A in GC through its effects on other factors, its related proteins themselves may also be regulated by non-coding RNAs and play a role in GC. Yan et al. (64) showed that the lncRNA LINC00470 is highly expressed in GC tissues. With involvement of the reader protein YTHDF2, the lncRNA LINC00470 affects the stability of PTEN by regulating the expression of METTL3, and this lncRNA also affects the proliferation, migration and invasion ability of GC cells. He et al. (82) confirmed that METTL3 affects the proliferation and apoptosis of GC cells by regulating SEC62, even though METTL3 itself is inhibited by miR-4429. These experiments all indicate that METTL3 expression is increased in GC but through different pathways, which indicates that the same m6A-related protein might play a role in the same tumor via different mechanisms. m6A is involved in the occurrence and development of GC and also plays an important role in the chemotherapy resistance of GC. METTL3 increases the stability of ARHGAP5 mRNA through the m6A modification and thereby causes drug resistance (66).

### Pancreatic Cancer

The role of m6A in pancreatic cancer has also been probed. Studies have found that both the mRNA and protein levels of YTHDF2 in pancreatic cancer tissues are significantly higher than those in paratumor tissues, and the expression of YTHDF2 tends to increase as the stage of the disease advances (83). Interestingly, this study found that YTHDF2 plays a different role in the proliferation, invasion, metastasis and EMT of pancreatic cancer cells. The knockout of YTHDF2 inhibits the proliferative ability of pancreatic cancer cells, although the invasive and metastatic abilities and EMT are enhanced, possibly due to different modes of action. Nonetheless, the study had an obvious deficiency, namely, a lack of clinical specimens. The analysis of the clinical relationship between YTHDF2 and pancreatic cancer was based only on a database analysis without any actual verification, and no *in vivo* studies were performed. The role of the m6A demethylase ALKBH5 in pancreatic cancer has also been reported (84): the mRNA level of m6A in pancreatic cancer tissues is significantly increased due to a decrease in the demethylase ALKBH5. ALKBH5 acts as a tumor suppressor *in vitro* and *in vivo* in pancreatic cancer and can inhibit its growth and metastasis by targeting PER1. Moreover, Zhang et al. (85) found that the expression of miR-25-3p is significantly higher in tumor tissues than in adjacent tissues from patients with pancreatic cancer who smoke and that a higher expression was associated with a worse prognosis. This phenomenon may be related to m6A, i.e., cigarette smoke condensate (CSC)-induced promoter hypomethylation upregulates METTL3 expression, and METTL3 promotes the maturation of miR-25-3p by the m6A modification. In short, smoking plays a role in the development and progression of pancreatic cancer through the METTL3/miR-25-3p/PHLPP2/AKT regulatory axis. This study is very interesting because it links smoking with m6A, which not only highlights new directions for pancreatic cancer treatment but also provides evidence that smoking is a risk factor for pancreatic cancer. m6A can influence not only the occurrence and metastasis of pancreatic cancer but also its resistance to drugs (86). The knockdown of METTL3 in pancreatic cancer cell lines increases the sensitivity of the cells to gemcitabine, cisplatin and other drugs, even though the morphology and proliferative abilities of the cells did not change, providing a new potential target for the treatment of pancreatic cancer. However, this study has some shortcomings, such as too few cell types and no *in vivo* experiments. The study was only superficial and did not explain the mechanism through which METTL3 increases drug sensitivity. Further research is needed.

### CRC

In addition to liver, stomach and pancreatic cancers, the clinical characteristics of CRC are also related to m6A. As the major m6A methyltransferase, METTL3 plays an important role in CRC (87–89). Peng et al. (87) found abnormal m6A modification in CRC. In normal tissues, paracancerous tissues and tumor tissues, the expression level of m6A exhibits a gradually increasing trend, and this change is mainly caused by increased expression of METTL3. Both *in vivo* and *in vitro* experiments have shown that the downregulation and upregulation of METTL3 reduces and enhances the metastatic ability of CRC, respectively, and this regulation is exerted through the METTL3/miR-1246/SPRED2 axis. Another study reached a similar conclusion. Li et al. (88) found that the expression of METTLE3 is significantly increased in primary CRC tissues compared with adjacent normal tissues and that METTL3 is significantly elevated in corresponding lymph node and liver metastatic foci. Patients with high METTL3 expression experience worse chemotherapy effects and shorter overall survival (OS) and disease-free survival (DFS) durations. As an oncogene, METTL3 plays a role by maintaining SOX2 expression in CRC cells through an m6A-IGF2BP2-dependent mechanism. However, there are contrasting conclusions regarding the role of METTL3 as a tumor suppressor in CRC (89). This study found that METTL3 expression is significantly negatively correlated with the tumor size and metastasis but positively correlated with patient prognosis; that is, a higher METTL3 expression in tumor is related to a better prognosis. This study further demonstrated that METTL3 plays a tumor-suppressive role in the proliferation, migration and invasion of CRC cells through the p38/ERK pathway. The reasons for the opposite conclusions reached for the same tumor and the same m6A protein may be due to sample problems, different transcripts of METTL3, or different risk factors that cause disease warrant further study. Nishizawa et al. found that YTHDF1 is an independent prognostic factor for CRC (90). YTHDF1 expression is significantly higher in CRC tissues than in normal tissues, and the YTHDF1 level is positively correlated with the depth of tumor invasion, lymph node metastasis, and clinical stage. The knockdown of YTHDF1 inhibits the proliferation of CRC cells and increases the sensitivity to oxaliplatin and other chemotherapeutic agents. However, this study had many limitations, such as too few clinical samples, incomprehensive experiments, no *in vivo* experiments, and no drug-resistant cell lines. Although YTHDF1 is an m6A reader, its downstream target was not identified in the study; that is, this target was not studied from the perspective of m6A. YTHDC2 also plays an important role in the metastasis of colon cancer (52). One study found a positive correlation between YTHDC2 and the clinical stage of colon cancer, including metastasis. The knockdown of YTHDC2 in colon cancer cells reduces the expression of metastasis-related proteins such as HIF-1α and inhibits tumor metastasis. The levels of eIF3 are also significantly higher in colon cancer tissues than in paratumor tissues, and the expression level of eIF3 is positively correlated with the tumor size, lymph node metastasis, distant metastasis, and vascular invasion, among others. The downregulation of eIF3 in colon cancer cells can inhibit cell proliferation and promote apoptosis (91). It should be noted that this study only collected data from patients at a single hospital for 2 years, and its limitations include a lack of representativeness, no *in vivo* experiments, a small number of experimental cells, and no mechanistic investigation. Therefore, this study has no practical reference value. The WNT signaling pathway plays a pivotal role in CRC, and m6A also reportedly plays a role through this pathway (67, 68, 92, 93). Song et al. (92) found that m6A is related to the occurrence of CRC, which is caused by METTL3 and related to the WNT signaling pathway. Increases in the expression of β-catenin increases METTL3 expression by inhibiting miR-455-3p and further increases the m6A modification of HSF1 to promote protein translation. The upregulation of β-catenin increases the HSF1 protein levels by promoting protein translation with no change in the mRNA levels or the protein half-life. In this study, the WNT signaling pathway, miRNAs and m6A were combined, and their relationship was described. Bai et al. (68) found that YTHDF1 can affect the stem cell-like activity of CRC cells and the proliferation ability of cells by affecting the cell cycle. A reduction in the expression of YTHDF1 will arrest cells at the G1 phase. Interestingly, the study found that YTHDF1 works by influencing the Wnt/β-catenin pathway rather than by being affected. However, this study also has some shortcomings, such as a small clinical sample size and many results from a database analysis. In addition to YTHDF1, YTHDF2 also affects the growth of CRC through the Wnt/β-catenin pathway (93). In CRC cells, increased protein levels of YTHDF2 are caused by decreased miR-6125. GSK3β mRNA can exhibit the M6A modification by increasing the YTHDF2 protein levels, which reduces the GSK3β mRNA stability and facilitates its degradation, and the levels of GSK3β protein and phosphorylated β-catenin are decreased. Abnormal accumulation of β-catenin activates cyclin D1 and thereby promotes CRC cell proliferation and tumor progression. This regulatory axis can be summarized as the miR-6125/YTHDF2/GSK3β/β-catenin/cyclin D1 regulatory pathway. In addition to clarifying the role of YTHDF2 in CRC, this study also noted the m6A modification site of GSK3β. m6A combined with the WNT signaling pathway not only plays a role in the occurrence and development of CRC but also leads to increases in the stemness of CRC cells and chemoresistance (67). Liu et al. found that Sec62 expression is increased in CRC. Elevated Sec62 binds to β-catenin to inhibit its degradation and enhance WNT signaling, which leads to increases in the stemness and chemoresistance of CRC cells. The elevation of Sec62 is caused by increased METTL3 expression. The m6A modification increases the stability of Sec62 mRNA.

## m6A and Non-neoplastic Diseases of the Gastrointestinal Tract

In addition to playing a pivotal role in digestive system tumors, m6A also plays an important role in some non-tumor diseases. Wu et al. found that circRNAs are involved in important processes, such as the regulation of autophagy and protein digestion, in mouse models of severe acute pancreatitis. The observed changes in circRNA function are caused by increased ALKBH5 expression and decreased m6A levels (94). HBV infection is not only the main cause of chronic hepatitis but also closely related to cirrhosis and liver cancer (95). A recent study showed that the m6A modification of YTHDF2 and YTHDF3 can regulate the HBV lifecycle by decreasing HBV RNA stability and HBV protein expression or promoting the reverse transcription of pregenomic RNA (96). It has also been reported that the m6A modification can regulate the lifecycle of HCV, that METTL3 and METTL14 can negatively regulate HCV infection, and that FTO can positively regulate HCV infection. In addition, m6A does not regulate HCV translation or RNA replication but can regulate the production of infectious virus particles, which this process is negatively regulated by YTHDF proteins (97). This finding was also confirmed by Kim et al. (98). Although YTHDF proteins do not affect HCV translation and replication, YTHDC2 may be involved in the secondary structure of the HCV IRES region through its helicase domain to promote HCV IRES-mediated translation.

In addition to its roles in severe acute pancreatitis and viral hepatitis, the role of m6A in intestinal non-tumor diseases has also been studied. Wang et al. (99) found that ALKBH5 upregulates TAGLN expression by demethylating TAGLN mRNA and then inhibits the proliferation and migration of enteric neural crest cells, which results in promotion of the occurrence of Hirschsprung's disease. Lu et al. constructed a new model of METTL14 deletion-induced spontaneous colitis in mice and confirmed that METTL14 deficiency impairs the ability of naïve T cells to induce induced T_reg_ cells and thus promote the development of colitis (100). By studying the regulation of m6A on T cells, Li et al. found that METTL3 in mouse T cells could regulate T cell homeostasis by targeting the IL-7/STAT5/SOCS pathway and that the deletion of METTL3 destroys T cell homeostasis and differentiation. Naïve METTL3-deficient T cells are unable to undergo homeostatic expansion and remain significantly naïve for up to 12 weeks to prevent colitis in a lymphopenic mouse model of adoptive transfer (101). ALKBH5 plays an important role in gastric intestinal metaplasia caused by bile acid reflux (102). On the one hand, ALKBH5 abolishes YTHDF2-dependent mRNA degradation by the demethylation of ZNF333 mRNA and increases the expression of ZNF333, and on the other hand, ALKBH5 activates CDX2 by targeting the ZNF333/CYLD axis and activating NF-κB signaling. This study suggests that ALKBH5 is a promising therapeutic target for gastrointestinal metaplasia caused by bile reflux. Although the role of m6A in non-tumor diseases of the gastrointestinal tract has been studied, the research progress is limited mainly due to the following two reasons: too few types of diseases have been studied and the mechanism is unclear. The reasons for this phenomenon may be the following: First, the study of gastrointestinal non-tumor diseases mainly relies on the establishment of animal models, and the collection of sufficient clinical samples for analysis and cell experiments is difficult. Second, many diseases can be diagnosed by blood and imaging tests alone without any need for genetic testing. Third, most non-tumor diseases can be controlled or cured with medication or surgery alone.

## m6A and Small-Molecule Inhibitors

The ultimate purpose of studying the role of m6A in tumors is to provide a new treatment direction. Many studies have reported small-molecule inhibitors of m6A, which mainly target METTL3 (103) and, in particular, FTO. A recent study reported that the small-molecule inhibitor STM2457 reduces the growth of acute myeloid leukemia (AML) and increases its differentiation and apoptosis (103). The pharmacological inhibition of METTL3 *in vivo* leads to impaired implantation and prolonged survival in a variety of mouse models of AML, particularly by targeting key stem cell subpopulations of AML. Overall, the small-molecule inhibition of METTL3 is not conducive to the maintenance of AML and exerts no significant or lasting effect on normal hematopoietic function. Qiao et al. (104) found that CHTB acts as an inhibitor of FTO and can specifically bind to it to increase the cellular m6A levels. However, the cells used in this study were not tumor cells, and whether the characteristics of the cells changed after the inhibition of FTO with CHTB is unclear. The natural compounds radicol and nafamostat mesilate inhibit FTO activity (105, 106), but these studies were not clinical studies and only demonstrated activity against FTO without any *in vivo* or *in vitro* analysis. It has also been reported that entacapone can directly bind to FTO to inhibit its activity (107). In mice with diet-induced obesity, the administration of entacapone reduces the body weight and fasting glucose concentrations, and entacapone affects gluconeogenesis in the liver and thermogenesis in adipose tissues of mice through the FTO/FOXO1 regulatory axis. Although this study was a clinical study, it was still a non-tumor study, and the results cannot be directly applied for the treatment of tumors, particularly gastrointestinal tumors. In addition to non-clinical studies, clinical studies of FTO inhibitors have been conducted. One study showed that meclofenamic acid can promote cisplatin-induced acute kidney injury by inhibiting FTO (108). *In vivo* and *in vitro* experiments have fully demonstrated that meclofenamic acid can affect the level of m6A by inhibiting FTO and increase the p53 mRNA and protein expression levels, which aggravates the acute kidney injury induced by cisplatin. Although meclofenamic acid does not affect the m6A level of mRNA, it can help clinicians avoid aggravation of but not treat the disease. Some studies have also investigated FTO inhibitors for tumors. Huang et al. (109) developed two promising FTO inhibitors, namely, FB23 and FB23-2, which can directly bind to FTO, selectively inhibit its m6A demethylase activity (particularly FB23-2), and thus play a role in AML. This study found that FB23-2 exhibits high selectivity for FTO and can significantly inhibit FTO expression to promote myeloid differentiation and apoptosis. *In vivo* experiments have also indicated that FB23-2 inhibits the progression of leukemia and improves the survival of leukemic mice. Most importantly, FB23-2 exhibits no toxicity or side effects in mice.

## Conclusions and Outlook

**Figure 2:**
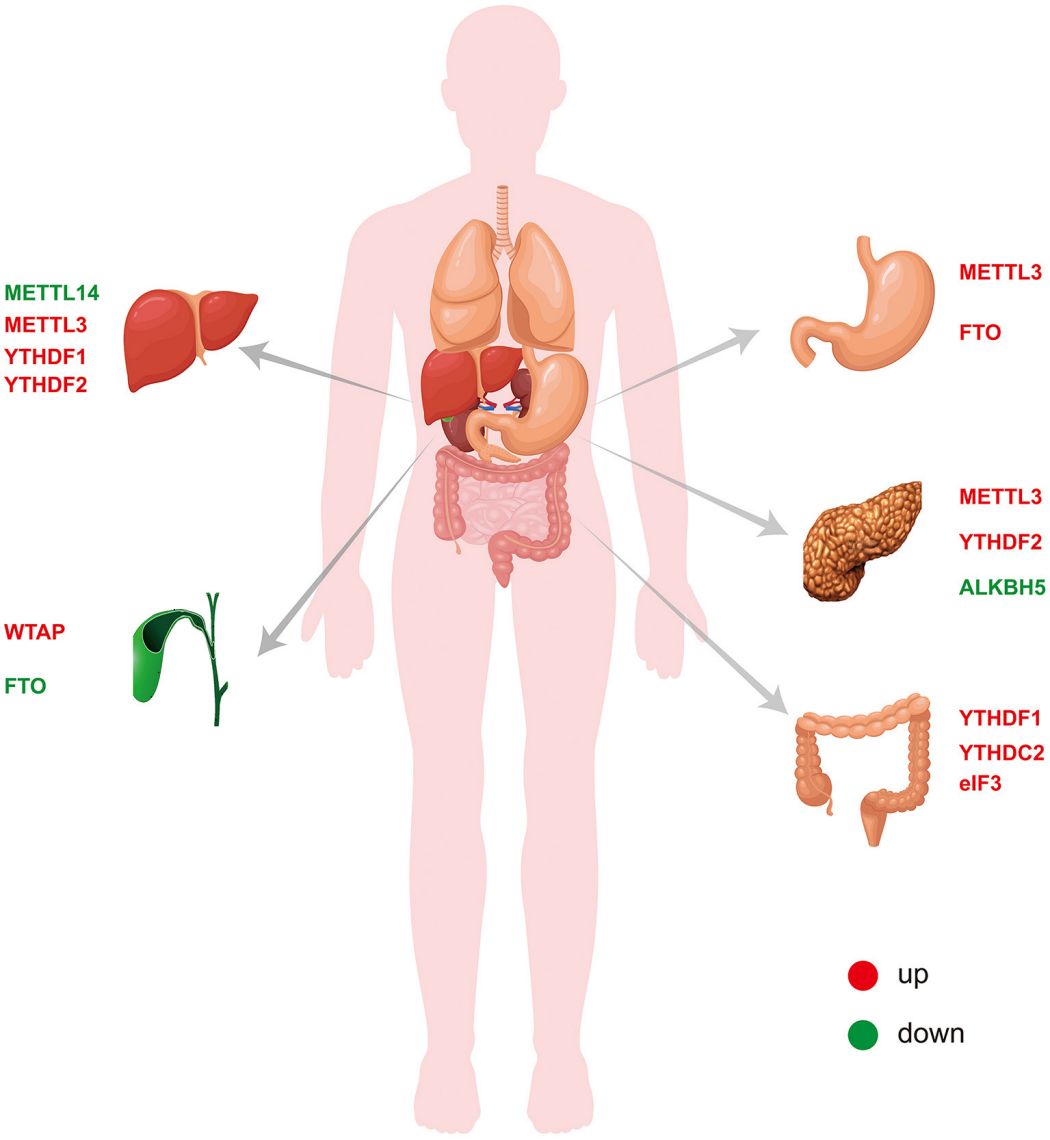
Expression of m6A-related proteins in digestive system tumors.

The m6A modification, which is the most abundant epigenetic modification of mRNA in higher eukaryotes, is a process through which the genetic information of an organism can be changed without altering the genetic sequence. Similar to DNA methylation, mRNA m6A modification is a reversible and dynamic process that is performed by the interaction of writers, erasers and readers. Through methylation and demethylation, m6A can not only participate in the metabolic process of mRNA but also further affect the occurrence and development of tumors. Digestive tract tumors are the most common tumors in humans, and many studies have found that m6A plays an important role. In this review, we summarize the roles and mechanisms of the m6A modification in liver cancer (70–73), CCA (75, 76), GC (63–66, 77–82), pancreatic cancer (83–86) and CRC (87–91) (Table 1 and Figure 2). These studies serve as good bases for the diagnosis and treatment of the corresponding tumors. However, the overall role of m6A in tumors remains not well-understood. In liver cancer, changes in the m6A levels are caused by increased METTL3 and/or METTL14 expression. In addition to writer proteins, reader proteins are altered in liver cancer, but their role is unclear (72, 73). For example, although many studies have confirmed that METTL3 expression is increased in GC, it plays different roles (63, 78). Thus, further details need to be revealed. Studies of the same tumor conducted by different researchers revealed that the m6A-related proteins METTL3 and FTO were important, although different results were obtained (77, 78). One study found that METTL3 but not FTO is significantly increased in cancer tissues, whereas the other study found that both were significantly increased. In CRC, different researchers even obtained completely opposite results for METTL3 expression (87–89). Moreover, the role of m6A in CCA remains unknown. Moreover, all the studies on m6A have many deficiencies, such as a lack of clinical samples, no *in vivo* or *in vitro* experiments, the inclusion of only superficial observations, no mechanistic investigation, and an inability to provide practical help for clinical application. Chemotherapy resistance has always been an important reason for poor therapeutic effects in cancer treatment, and the discovery of m6A provides a new direction to reduce chemotherapy resistance. Despite many studies in this direction, there remain limitations, and thus, the results do not provide any practical evidence for clinic application. In addition to tumors, m6A has also been studied in gastrointestinal non-tumor diseases, but the related research progress has been limited by the limitations of research methods and practical applications. The emergence of small-molecule inhibitors of m6A has provided new hope for the treatment of diseases, and many studies have explored the discovery and effects of such inhibitors. At present, the identified inhibitors mainly target two enzymes, METTL3 and in particular, FTO. Moreover, many inhibitors have only been shown to inhibit FTO, but their specific biological effects have not been explained. To date, the research on small-molecule inhibitors of m6A in tumors remains limited to AML, and studies on digestive tract tumors have not been reported. There are many difficulties regarding the application of small-molecule inhibitors of m6A to gastrointestinal malignancies. First, drugs that specifically bind to m6A-related proteins need to be identified or synthesized. Second, these drugs must act on m6A-related proteins by inhibiting their activity and not through other effects. Third, these small-molecule inhibitors must be more effective when used alone or in combination with clinically available antitumor agents. There is still a long way to go before small-molecule inhibitors can be used for the treatment of gastrointestinal malignancies. All of these problems need to be further addressed. The role of m6A in tumors is unquestionable, but there remain many issues to be resolved regarding not only gastrointestinal tumors but also other tumors. Further research will provide new directions for the diagnosis and treatment of tumors.

**Table 1:** The roles and mechanisms of the m6A modification in digestive system tumors.

**Cancer**	**m6A Regulator**	**Type**	**Role**	**Change**	**Mechanism**	**References**
HCC	METTL14	Writer	Suppressor	Down	METTL14 downregulates micRNA126 by reacting with DGCR8.	(71)
	METTL3	Writer	Promoter	Up	METTL3 represses SOCS2 expression through an m6A/YTHDF2-dependent mechanism.	(70)
	YTHDF2	Reader	Promoter	Up	miR-145 modulates the m6A levels by targeting the 3'-UTR of YTHDF2 mRNA.	(72)
	YTHDF1	Reader	Promoter	Up	YTHDF1 regulates HCC cell cycle progression and metabolism.	(73)
CCA	WTAP	Writer	Promoter	Up	WTAP induces the expression of MMP7, MMP28, cathepsin H and Muc1.	(75)
	FTO	Eraser	Suppressor	Down	FTO regulates ICC progression through multiple key oncogenes and suppressors.	(76)
GC	METTL3	Writer	Promoter	Up	The METTL3-mediated m6A modification of HDGF mRNA promotes GC progression.	(65)
	FTO	Eraser	Promoter	Up	NA	(77)
	METTL3	Writer	Promoter	Up	METTL3 regulates the MYC pathway.	(78)
	METTL3	Writer	Promoter	Up	METTL3 affects the proliferation and migration abilities of GC cells by regulating the expression level of GFI-1 and EMT.	(79)
	METTL3	Writer	Promoter	Up	METTL3 regulates BATF2 mRNA and represses its expression.	(63)
	METTL3	Writer	Promoter	Up	METTL3 promotes the proliferation, migration and invasion of GC cells by activating the Akt pathway.	(80)
	METTL3	Writer	Promoter	Up	METTL3 enhances ZMYM1 mRNA expression through the m6A/HuR-dependent pathway.	(81)
	METTL3	Writer	Promoter	Up	LINC00470 promotes GC progression through the METTL3/PTEN axis.	(64)
	METTL3	Writer	Promoter	Up	miR-4429 inhibits GC progression through the METTL3/SEC62 axis.	(82)
	METTL3	Writer	Promoter	Up	ARHGAP5-AS1 recruits METTL3 for the m6A modification of ARHGAP5 mRNA.	(66)
Pancreatic cancer	YTHDF2	Reader	Promoter	Up	YTHDF2 regulates EMT probably via YAP signaling.	(83)
	ALKBH5	Eraser	Suppressor	Down	ALKBH5 loss downregulates the PER1 mRNA levels in a m6A/YTHDF2-dependent manner.	(84)
	METTL3	Writer	Promoter	UP	Cigarette smoke promotes the development and progression of pancreatic cancer via the METTL3/miR-25-3p/PHLPP2/AKT regulatory axis.	(85)
	METTL3	Writer	Promoter	Up	METTL3 influences the sensitivity of pancreatic cancer cells to anticancer reagents *via* a ubiquitin-dependent process, RNA splicing and the regulation of cellular processes.	(86)
CRC	METTL3	Writer	Promoter	Up	METTL3 promotes metastasis of CRC via the miR-1246/SPRED2/MAPK signaling pathway.	(87)
	METTL3	Writer	Promoter	Up	METTL3 facilitates CRC progression via a m6A/IGF2BP2-dependent mechanism.	(88)
	METTL3	Writer	Suppressor	Down	METTL3 suppresses CRC proliferation and migration through p38/ERK pathways.	(89)
	METTL3	Writer	Promoter	UP	β-catenin suppresses miR455-3p to increase the m6A modification of HSF1 mRNA.	(92)
	METTL3	Writer	Promoter	UP	m6A modification/Sec62/β-catenin molecular axis.	(67)
	YTHDF1	Reader	Promoter	Up	The oncogenic transcription factor c-Myc regulates YTHDF1 in CRC.	(90)
	YTHDF1	Reader	Promoter	Up	YTHDF1 inhibits Wnt/β-catenin pathway activity.	(68)
	YTHDF2	Reader	Promoter	Up	miR-6125/YTHDF2/GSK3β/β-catenin/cyclin D1 regulatory axis	(93)
Colon tumor	YTHDC2	Reader	Promoter	Up	YTHDC2 contributes to colon tumor metastasis by promoting the translation of HIF-1α.	(52)
	eIF3	Reader	Promoter	Up	NA	(91)

## Author Contributions

TC, WM, and YL involved in study design and drafting of the manuscript. TC and LA revised the manuscript. XZ, CH, MB, HM, CZ, WF, and LG reviewed and approved the manuscript. All authors contributed to the article and approved the submitted version.

## Funding

Health Industry Scientific Research Program of Gansu Province (No. GSWSKY2020-11); Lanzhou Chengguan District Science and Technology Plan Project (No. 2019JSCX0092); National Natural Science Foundation of China (No. 82060551); and Lanzhou Chengguan District Science and Technology Plan Project (No. 2019RCCX0038).

## Conflict of Interest

The authors declare that the research was conducted in the absence of any commercial or financial relationships that could be construed as a potential conflict of interest.

## Publisher's Note

All claims expressed in this article are solely those of the authors and do not necessarily represent those of their affiliated organizations, or those of the publisher, the editors and the reviewers. Any product that may be evaluated in this article, or claim that may be made by its manufacturer, is not guaranteed or endorsed by the publisher.
